# Dr. Willis M. Wells

**Published:** 1912-05

**Authors:** 


Dr. Willis M. Wells, died at his home in Fulton, N. Y., March 30, 1912, aged 61. Death was due to cerebral haemorrhage. He was graduated at the University of Vermont, 1874. He had been President of the Oswego County Medical Society, of the Fulton Academy of Medicine and had held other offices of honor.

